# Fermented Cassava Residue Meal Improves Meat Quality by Regulating Muscle Fiber and Enhancing Lipid Metabolism in Huanjiang Mini-Pigs

**DOI:** 10.3390/ani15020177

**Published:** 2025-01-10

**Authors:** Huijiao Jiang, Md. Abul Kalam Azad, Qian Zhu, Hengjia Ni, Xiangfeng Kong

**Affiliations:** 1 CAS Key Laboratory of Agro-Ecological Process in Subtropical Region, Hunan Provincial Key Laboratory of Animal Nutritional Physiology and Metabolic Processes, National Engineering Laboratory for Pollution Control and Waste Utilization in Livestock and Poultry Production, Institute of Subtropical Agriculture, Chinese Academy of Sciences, Changsha 410125, China; ppjhj2035@163.com (H.J.); azadmak@isa.ac.cn (M.A.K.A.); zhuqian@isa.ac.cn (Q.Z.); nihengjia@isa.ac.cn (H.N.); 2 College of Advanced Agricultural Sciences, University of Chinese Academy of Sciences, Beijing 100049, China

**Keywords:** alternative feed resources, meat quality traits, muscle fiber formation, pigs, serum biochemistry

## Abstract

This research investigated the effects of cassava residue meal (CRM) and fermented CRM (FCRM) on the growth performance, serum lipid indicators, carcass traits, and meat quality of Huanjiang mini-pigs. One hundred twenty Huanjiang mini-pigs with similar BW (body weight, 8.85 ± 0.64 kg) were divided into three dietary treatment groups: the CON group with a basal diet; the CRM group with a diet containing 5% CRM; and the FCRM group with a diet containing 5% FCRM. The feeding trial was conducted for 30 days. The findings revealed that dietary CRM and FCRM did not influence the growth performance and diarrhea rate of Huanjiang mini-pigs (*p* > 0.05). Dietary FCRM supplementation increased serum total cholesterol level compared to the CON group while decreasing serum cholinesterase level compared to the CON and CRM groups (*p* < 0.05). The level of serum low-density lipoprotein-cholesterol was higher (*p* < 0.05) in the CRM and FCRM groups in regard to the CON group. In addition, CRM and FCRM supplementation reduced (*p* < 0.05) the backfat thickness and carcass yield of Huanjiang mini-pigs. The meat quality analysis showed that dietary CRM and FCRM improved the meat quality by increasing the intramuscular fat content and redness (a*) value and decreasing the lightness (L*) value. Moreover, the *MyHC-IIx* expression in the *longissimus thoracis* (LT) muscle was upregulated (*p* < 0.05) in the CRM group, while *MYHC-IIb* expression displayed an increasing trend (*p* = 0.076) in the FCRM group relative to the CON group. The fatty acid composition in the LT muscle also revealed that the C20:0 level was lower in the CRM and FCRM groups, while the ∑SFA and ∑SFA/∑UFA were reduced (*p* < 0.05) in the FCRM group relative to the CON group. In summary, dietary CRM and FCRM supplementation improved the meat quality of Huanjiang mini-pigs without affecting growth performance and diarrhea rate. Notably, FCRM exhibited better effects on meat quality than CRM.

## Simple Summary

The use of agricultural by-products in livestock production has gained increasing interest due to their rich nutritional value and bioactive compounds. However, various anti-nutritional factors limit their application as feed additives in monogastric animals. The anti-nutritional factors of agricultural by-products can be minimized through microbial fermentation. This study investigated the effects of cassava residue meal and fermented cassava residue meal and found that these feed additives were beneficial for the meat quality of Huanjiang mini-pigs without affecting their growth performance.

## 1. Introduction

The livestock industry in China has undergone significant changes in recent decades, leading to profound implications for both domestic and global food security [1]. China produces more than 50% of global pork production and is the largest pig-producing country globally. The swine industry has become the most important economic source of agriculture in China due to the large-scale and intensive industry. However, swine industries in China are currently facing a shortage of high-quality feed resources, particularly corn and soybean. These feed resources are highly compatible with human food resources, for which China is mostly dependent on imports, which greatly restricts the sustainable development of swine industries [2]. Therefore, the development of non-conventional feeds is imperative in mitigating the conflict between human and animal food and fostering the growth of the livestock industry.

Cassava (*Manihot esculenta* Crantz.) can survive in arid and low-fertility acidic soils and is mainly distributed in Africa, Asia, South America, and other tropical and subtropical countries [3,4]. Cassava is one of the staple food sources in many countries. Additionally, cassava has wide applications, such as starch production, bioethanol manufacturing, and the application of other valuable bio-products like medicines, biopolymers, and animal feed [5,6]. In China, Guangxi is the main region for cassava cultivation, accounting for 60% of the total annual cultivation area of the country [7]. The favorable geographical and environmental conditions in Guangxi make it superior to other regions in China regarding cassava yield and quality. However, there are still challenges to the development of the cassava industry, including the management of starch production by-products.

Significant amounts of cassava residue meal (CRM) are generated during the starch production process, most of which are unused and cause environmental pollution. Thus, the appropriate utilization of CRM as animal feed is crucial in addressing food security concerns. However, CRM contains lower content of protein and essential amino acids, thereby limiting its use in animal diets due to the cost requirements of additional protein sources in cassava [8]. Furthermore, CRM is also characterized by poor palatability and the potential formation of toxic cyanogenic compounds [9]. Previous studies have shown that fermentation offers advantages in enhancing nutrient bioavailability (e.g., reducing toxicity and improving palatability) and nutritional value of non-conventional feeds [10,11]. The inclusion of fermented CRM (FCRM) in animal diets has been found to raise the growth performance of livestock, reduce production costs, and improve overall benefits [12,13]. Therefore, using CRM as animal feed through microbial fermentation not only benefits economically but also contributes to environmental protection.

The Huanjiang mini-pig represents a key protected mini-pig breed in China. The current feeding management practice, limited feed formulation interest for a specific pig breed, and the inherent characteristics of the pig species lead to the slower growth and lower production rate of Huanjiang mini-pigs. Research evidence indicates that domestic pigs have better adaptability to the local environment and roughage feeding tolerance due to their distinct genotype characteristics from commercial pigs [14]. However, the application of agricultural by-products as animal feed and the utilization of non-conventional feed resources for domestic pig industry development still need to be explored. Moreover, naturally present bioactive compounds in agricultural by-products, as well as nutritional enrichment through microbial fermentation, could influence the growth performance and thus influence the meat quality of animals. Therefore, considering the genetic advantages of domestic pigs and available local feed resources, we hypothesized that CRM and FCRM would be potential non-conventional feed resources, which may influence the productivity and meat quality of domestic pigs. Therefore, the present study selected Huanjiang mini-pigs as experimental animals to explore the impacts of dietary CRM and FCRM supplementation on growth performance, diarrhea rate, serum lipid indicators, and meat quality. The outcomes will provide a guiding significance for the utilization of non-conventional feed resources for pig production.

## 2. Materials and Methods

### 2.1. Preparation and Fermentation of CRM

The CRM (containing 16.13 MJ/kg gross energy, 2.73% crude protein, 37.73% neutral detergent fiber, and 2.52% minerals) was sourced from Du’an Honghe Starch Co., Ltd. (Hechi, China). Briefly, the integral CRM was sun-dried to reduce the water content. Then, the final CRM product, which contained about 40% water, was stored in plastic bags at 4 °C until further processing. The FCRM was prepared as described in our previous study [15]. Briefly, 200 kg of CRM and 20 kg of corn flour were properly mixed, and then 0.2 kg of bacterial fermentation liquid broth (containing *Lactobacillus plantarum* 1.0 × 10^8^, *Saccharomyces cerevisiae* 0.2 × 10^8^, and *Bacillus subtilis* 0.2 × 10^8^) was properly mixed with the basal feed. Afterward, the fermentation mixture was packed in a 15 L sealed plastic bag with 5 kg of mixture per bag at 27–30 °C for seven days. The CRM and FCRM were sun-dried and used for feed. The liquid broth for fermentation was supplied by Hunan Lifeng Biotechnology Co., Ltd. (Changsha, China). The chemical composition of FCRM contained 16.51 MJ/kg gross energy, 4.79% crude protein, 29.39% neutral detergent fiber, and 7.81% minerals.

### 2.2. Animals, Diets, and Housing Management

Twenty pregnant Huanjiang mini-sows with 3–5 parity were selected, and after natural delivery within a week, 120 piglets were selected from 20 litters as experimental animals. On 60 days of age, based on their average BW (8.85 ± 0.64 kg), piglets were selected and randomly allotted to one of three groups, with eight replicates (including four pens of males and four pens of females) in each group and five piglets per replicate. The control (CON) group pigs were fed a basal diet, while the CRM group pigs were fed a diet containing 5% CRM, and the FCRM group pigs were fed a diet containing 5% FCRM for 30 days. The CRM and FCRM were homogenously mixed with the basal diet before feeding. All piglets were housed in pens (2.0 × 3.0 m) equipped with a water nipple and a single-hole feeder in the same barn. The experimental animals were reared in a controlled room at a constant temperature maintained at 23–25 °C with relative humidity at 60 ± 5%. All experimental animals had ad libitum access to food and water during the trial. The basal diet was prepared in accordance with the nutrient requirements for 10 kg pigs recommended by the Chinese local swine nutrient requirements (NY/T65-2004; Table 1) [16]. All the animals for this trial were in good health conditions. Prior to the formal feeding trial, the experimental animals had no gastrointestinal diseases or any antibiotic exposure.

### 2.3. Determination of Growth Performance and Diarrhea Rate

The initial BW and final BW of pigs were measured, and the average daily gain (ADG) was calculated. The feed intake per pen was recorded daily to calculate the average daily feed intake (ADFI). The ratio of feed intake and weight gain (F/G) was calculated. The diarrheal conditions of pigs per pen were recorded every morning. The fecal score for the diarrheal condition was evaluated as follows: 0, no diarrhea (normal and firm feces); 1, mild diarrhea (pasty feces); 2, moderate diarrhea (unformed and moderately fluid feces); 3, severe diarrhea (very watery and frothy diarrhea). The diarrhea rate (%) was determined using a previously described equation [17].

Diarrhea rate (%) = total times of diarrhea/(number of piglets × days of experiment) × 100.

### 2.4. Sampling

On day 30 of the trial, eight piglets per group (half males and half females) within the average BW of the group were chosen for sampling. Approximately 5 mL of blood samples were drawn from the jugular vein of each selected piglet to obtain serum by centrifuging for 10 min at 3000× *g* and 4 °C. The serum samples were immediately taken into a new EP tube and placed at −80 °C for further analyses of lipid profile. The piglets were euthanized under standard commercial protocols and exsanguination. Subsequently, carcasses were split longitudinally after removing the head, legs, tail, and viscera to determine carcass traits and meat quality. *Longissimus thoracis* (LT) muscles were then harvested between the ninth and tenth ribs and placed in plastic bags at −20 °C to determine fatty acid composition. Additionally, approximately 2 g of LT muscle from each piglet was harvested, immediately frozen in liquid nitrogen, and placed at −80 °C for gene expression analyses.

### 2.5. Determination of Carcass Traits

Each carcass was separated (left side) into the skin, lean meat, fat, and bone following the Chinese pig-raising industry standards (GB8467-87) [18]. The weight of carcass, lean meat, fat, and bone was recorded to determine the percentage of live BW [tissue weight × 2 (kg)/BW (kg) × 100]. A Vernier caliper was used to determine the backfat thickness (between the sixth and seventh ribs) of pigs. The cross-sectional height and width measurement of the LT muscle were taken with a Vernier caliper to determine the loin-eye muscle area (height × width × 0.7).

### 2.6. Determination of Serum Lipid Indicators

The lipid indicators in serum, including triglyceride (TG), total cholesterol (TC), high-density lipoprotein-cholesterol (HDL-C), low-density lipoprotein-cholesterol (LDL-C), and choline esterase (CHE) levels, were determined with commercial kits (Roche, Basel, Switzerland) and a biochemical analyzer (Roche, Cobas c311; Basel, Switzerland).

### 2.7. Meat Quality Analysis

The pH values at 45 min (pH_45 min_) and 24 h (pH_24 h_) post-mortem were recorded with a laboratory pH meter (pH Star, Matthaus, Germany). Meat color of L* (lightness), a* (redness), and b* (yellowness) values were measured with a color analyzer instrument (Minolta CR-410; Kinica Minolta Sensing Inc., Tokyo, Japan). The LT samples preserved at 4 °C were transferred to individual vacuum polyethylene bags and cooked at 75 °C for 30 min. Then, the samples were cooled until they reached the ambient temperature, dried with an absorbent paper, and measured the weight to determine the cooking yield using a previously described method [19].

### 2.8. Determination of Intramuscular Fat Content and Fatty Acid Composition in Muscle

Preserved LT muscle samples were cut into thin slices after weighing, dried using a vacuum-freeze drying method (at 20 ± 5 Pa and −45 ± 5 °C for 72 h), and ground into powder, as previously described by Zhu et al. [19]. The content of intramuscular fat (IMF) in the LT muscle was assessed with a Soxhlet extraction apparatus and the method described previously [20]. The medium-chain fatty acid (MCFA) and long-chain fatty acid (LCFA) composition in the LT muscle was measured by gas–liquid chromatography using a previously described method [21]. The fatty acid composition was expressed as a percentage of total fatty acids.

### 2.9. Analysis of Gene Expressions Related to Myosin Heavy Chain Isoform in Muscle

The total RNA from muscle samples was isolated with the AG RNAex Pro reagent extraction kits (Accurate Biology, Changsha, China), following the guidelines provided by the manufacturers. The obtained RNA concentration was determined with an RNA analyzer (NanoDrop ND-2000; Thermo Fisher Scientific, Waltham, MA, USA). The ratio of the absorption at 260/280 nm was used to check the purity of RNA, while agarose gel electrophoresis was utilized to detect the integrity of RNA. Approximately 1000 ng total RNA was converted into cDNA with the DNA eraser kits for quantitative PCR analysis (Accurate Biology), which was conducted on the PCR System (LightCycler R 480II; Roche, Basel, Switzerland) with a SYBR R Green Premix Pro Taq HS qPCR Kit (Accurate Biology). The RT-PCR cycling included 10 min of preliminary denaturation at 95 °C, then 40 cycles of denaturation at 95 °C for 15 s and annealing at 60 °C for 30 s, and the final extension at 72 °C for 30 s. The listed (in Table 2) target gene primers were sourced from Sangon Biotech Co., Ltd. (Shanghai, China). The expression of each gene was evaluated using the 2^−ΔΔCT^ method [22].

### 2.10. Statistical Analysis

All experimental data were analyzed using SPSS v26.0 (Chicago, IL, USA) with a one-way ANOVA after verifying the homogeneity of variances using Levene’s test. Differences among the three groups were evaluated using Tukey’s post hoc comparison. Experimental pens were used as the experimental unit for growth performance analyses, whereas individual experimental pigs were considered as the experimental unit for other analyses. All data are presented as means with their SEM. Differences among groups were considered significant if *p* < 0.05 and a trend if 0.05 ≤ *p* < 0.10.

## 3. Results

### 3.1. Effects of CRM and FCRM on the Growth Performance and Diarrhea Rate of Huanjiang Mini-Pigs

The impacts of CRM and FCRM on the growth performance and diarrhea rate of Huanjiang mini-pigs are listed in Table 3. There were no significant differences among different groups in the final BW, ADG, ADFI, F/G, and the diarrhea rate (*p* > 0.05).

### 3.2. Effects of CRM and FCRM on Serum Lipid Indicators of Huanjiang Mini-Pigs

The results of serum lipid indicators are presented in Table 4. Diets supplemented with FCRM elevated (*p* < 0.05) the serum TC level compared with the CON group. Diet supplemented with FCRM reduced (*p* < 0.05) the serum CHE level relative to the CON and CRM groups. Moreover, diets supplemented with CRM and FCRM increased the serum LDL-C level compared with the CON group (*p* < 0.05). However, dietary CRM and FCRM supplementation had no effects on serum TG and HDL-C levels (*p* > 0.05).

### 3.3. Effects of CRM and FCRM on Carcass Traits and Meat Quality of Huanjiang Mini-Pigs

As listed in Table 5, CRM and FCRM supplementation reduced (*p* < 0.05) carcass yield and backfat thickness of pigs in comparison with the CON group. However, there are no significant changes in the loin-eye area and the percentages of lean meat, fat, and bone of piglets (*p* > 0.05).

As shown in Table 6, the pH_24 h_ value displayed a decreasing trend (*p* = 0.090) in the CRM group with regard to the CON group. Dietary CRM and FCRM supplementation decreased L* value while increasing a* value in the LT muscle relative to those in the CON group. Moreover, the IMF content was higher (*p* < 0.05) in the CRM and FCRM groups relative to that in the CON group.

### 3.4. Effects of CRM and FCRM on the mRNA Expression of Gene in Muscle of Huanjiang Mini-Pigs

**Figure 1:**
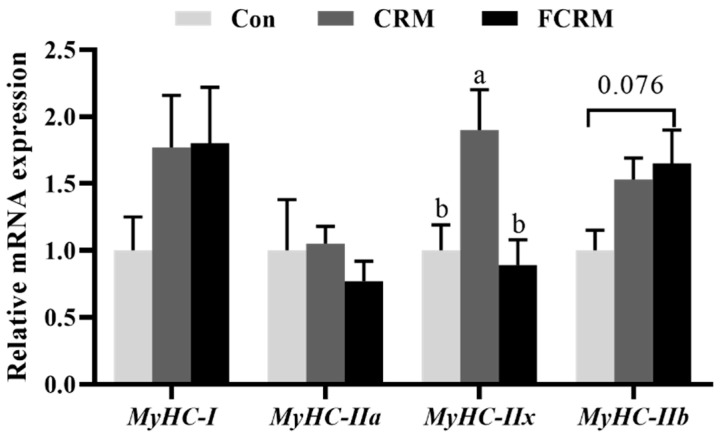
The mRNA expression of myosin heavy chain (*MyHC*) isoform genes in the *longissimus thoracis* muscle of Huanjiang mini-pigs. Data are presented as means with their SEM (*n* = 8). Mean values without a common lowercase letter are significantly different at *p* < 0.05. CON, control group; CRM, cassava residue meal group; FCRM, fermented cassava residue meal group; *MyHC*, myosin heavy chain.

As shown in Figure 1, the *MyHC-IIx* expression in the LT muscle was upregulated (*p* < 0.05) in the CRM group in comparison with the CON and FCRM groups. Additionally, diets supplemented with FCRM showed an increasing upregulating trend (*p* = 0.076) in the *MyHC-IIb* expression relative to that in the CON group. However, diets supplemented with CRM and FCRM did not affect (*p* > 0.05) the expression of *MyHC-I* and *MyHC-IIa* in the LT muscle of Huanjiang mini-pigs.

### 3.5. Effects of CRM and FCRM on Fatty Acid Composition in Muscle of Huanjiang Mini-Pigs

The fatty acid composition in the LT muscle of Huanjiang mini-pigs is presented in Table 7. The C20:0 content was reduced (*p* < 0.05) in the CRM and FCRM groups compared with the CON group. The sum of saturated fatty acid (∑SFA) and the ratio of ∑SFA to the sum of unsaturated fatty acid (∑UFA; ∑SFA/∑UFA) were reduced (*p* < 0.05) in the FCRM group relative to those in the CON group. Additionally, the sum of mono-unsaturated fatty acid (∑MUFA) displayed a decreasing trend (*p* = 0.083) in the CRM and FCRM groups with regard to the CON group.

## 4. Discussion

By-products generated during the processing of agricultural products have significant nutrients and bioactive substances, such as polysaccharides, proteins, vitamins, minerals, and flavonoids. However, these by-products are frequently disposed of improperly after processing, leading to potential environmental pollution. Moreover, anti-nutritional factors in agricultural by-products restrict their application in animal diets. Previous investigations have demonstrated that fermentation can mitigate these anti-nutritional factors by degrading the cellulose and lignin found in CRM, subsequently enhancing its nutritional value, digestibility, and palatability [23,24]. Thus, our study investigated the impacts of partial replacement of corn–soybean meal by CRM and FCRM on the growth performance, diarrhea rate, serum lipid indicators, carcass traits, and meat quality of Huanjiang mini-pigs. The findings indicated that dietary CRM and FCRM supplementation could improve the meat quality without affecting the growth performance and diarrhea rate of Huanjiang mini-pigs.

Microbial fermentation produces volatile aromas, stimulating animals’ sense of smell and influencing feed intake. However, the findings of the present study indicated that diets supplemented with CRM and FCRM did not influence the growth of Huanjiang mini-pigs. It can be postulated that the digestive capacity of weaned piglets may still be underdeveloped, rendering them unable to utilize the nutrients derived from the CRM and FCRM effectively. A recent study indicated that cassava residue as an amylopectin source in low-protein diets elevated feed intake and thereby influenced the growth performance of growing pigs, while long-term feeding caused diarrhea and reduced the growth of piglets, which might be associated with anti-nutritional factors presented in cassava residue [25]. Additionally, different levels of sundried cassava peels (20%, 30%, and 40%) meals did not affect the growth performance of growing pigs [26]. These differences may be due to genetic diversity and the growing phases of the pigs.

Serum lipid biomarkers, such as TG, TC, HDL-C, and LDL-C, serve as markers for the synthesis and decomposition of body fat [27]. Among these indicators, TG is the potential lipid indicator in the animal body and plays a pivotal role in energy supply and storage, while TC exists in all animal tissues [28]. LDL-C facilitates the transportation of TC synthesized by the liver to extrahepatic tissues, whereas HDL-C transports TC to the liver for metabolism and conversion into other substances. Consequently, HDL-C contributes to the maintenance of TC value stability within the body [29]. In our study, diets supplemented with CRM and FCRM significantly elevated serum LDL-C level. Additionally, dietary FCRM supplementation led to an increase in serum TC level, which may be attributed to the emulsification of cholesterol and its potential association with bile acid reabsorption [30]. Cholinesterases are a group of enzymes responsible for the hydrolysis of acetylcholine and other cholinesterases [31]. The two main forms of cholinesterases are acetylcholinesterase and butyrylcholinesterase. Although butyrylcholinesterase has been long considered a marker of nutritional and hepatic protein synthesis, its specific physiological function has only recently been clarified [31]. Our results showed that the serum CHE level in the FCRM group was significantly decreased compared to the other two groups, implying a potential impact on the regulation of blood glucose and insulin sensitivity [32]. This finding indicates that partial replacement of a corn–soybean diet by FCRM might have the potential risk for hepatocyte dysfunction.

Carcass traits and meat quality are the crucial indicators that are associated with flavor, juiciness, tenderness, and overall acceptability of the meat. Additionally, carcass weight is positively correlated with the pre-mortem BW of animals. Slaughter performance depends on the performance and growth of animal breeds, including dressing percentage, bone percentage, backfat thickness, loin-eye area, etc. [33]. In the present study, carcass yield and backfat thickness of Huanjiang mini-pigs were significantly decreased when fed with CRM and FCRM. Previous studies found that bioactive compounds, such as polyphenols, tannic acid, and flavonoids present in natural or fermented agricultural by-products, prevent obesity by several mechanisms, including decreased lipogenesis, stimulated lipolysis, and suppressed adipocyte differentiation in animals, thereby reducing backfat thickness and increasing lean production [34,35]. Moreover, probiotic bacteria, such as *Lactobacillus plantarum* and *Saccharomyces cerevisiae*, have been reported to exhibit lipolytic activity [36]. Therefore, the decrease in dressing percentage and backfat thickness in the present study may be due to the alteration of lipid metabolism by the bioactive compounds present in CRM or probiotic bacteria in FCRM; however, further investigations warrant elucidation of the exact mechanism.

After slaughter, pH value, flesh color, cooking yield, and meat tenderness are the important indicators to measure meat quality [37]. Meat color determines the consumer’s first impression of meat and is the most direct index used to judge the quality of meat. The higher redness (a*) value and lower lightness (L*) and yellowness (b*) values indicate high-quality meat [38]. In the present study, diets supplemented with CRM and FCRM significantly increased the a* value and reduced the L* value in the LT muscle, indicating that dietary CRM and FCRM supplementation improved the meat quality of Huanjiang mini-pigs.

Additionally, the nutritional value and sensory characteristics of meat can be directly affected by the nutrient composition of the muscular tissue, particularly the content of IMF. The elevated IMF content substantially enhances the muscle tenderness, juiciness, color, and flavor of meat. Moreover, meat taste is also influenced by the IMF content of the meat [39]. Our findings indicated that the IMF content was higher in the LT muscle of the CRM and FCRM groups, indicating an enhancement of the meat quality, which is consistent with the improved meat color. Several recent studies have indicated that conventional diets fermented with different probiotic bacteria, including *Enterococcus faecium*, *Bacillus subtilis*, and *Pediococcus pentosaceus*, increased the IMF contents, thereby improving meat color in finishing pigs [40,41]. A possible explanation is that probiotic bacteria species may play a crucial role because antimicrobial metabolites produced through these probiotic bacteria may be beneficial for improving meat quality through IMF deposition [41]. Therefore, the increased IMF content and meat color of pigs in the FCRM group may be influenced by the fermenting bacteria species; however, the explanation for the CRM diet still needs further investigation.

The fatty acid composition reflects the potential nutritional value of the muscle, including organoleptic and eating quality of meat [42]. Previous studies reported that the fatty acid profile of meat could be enhanced by elevating the UFA and reducing SFA content in muscles [19,43]. Studies have also reported that the higher intake of SFA is closely correlated with an increased risk of obesity and obesity-associated diseases, while replacing SFA with UFA (including MUFA and PUFA) can reduce the risk of coronary heart disease [27,44]. Previously, it has been reported that supplementation of natural and fermented herbs (*Artemisia capillaris* and *Acanthopanax senticosus*) reduced SFA and increased MUFA concentrations in the *longissimus dorsi* muscle of growing-finishing pigs [45]. Moreover, fermented agricultural by-products of pomegranate peel, *Ginkgo biloba* leaves, and licorice root decreased SCFA and PUFA and increased MUFA concentrations in *longissimus dorsi* muscle of grower-finisher pigs [35]. In our study, consistent with those findings, dietary CRM and FCRM supplementation decreased SFA (C20:0) content and tended to increase MUFA content, while FCRM decreased ∑SFA content and the ∑SFA/∑UFA in the LT muscle of Huanjiang mini-pigs. These findings indicate that the bioavailability of natural bioactive compounds in dietary CRM and FCRM can possibly modify the fatty acid composition in the LT muscle of pigs by preventing the oxidation of UFA [35,46], thereby reducing the adverse health effects associated with FA composition in meat.

The characteristics of muscle fiber play a crucial role in determining muscle quality and are closely linked to cooking yield, cooking loss, and shear force [47]. During the early growth stage of animals, muscle fiber types undergo transformations influenced by physiological stage, nutrient level, and other factors, which significantly influence meat quality. The expression of *MyHC* polymorphism genes is commonly used to classify muscle fiber types in skeletal muscle [48]. Notably, adult mammals possess four MyHC isomers, including types I, IIa, IIx, and IIb, which correspond to slow contraction oxidation, fast contraction oxidation, fast contraction glycolysis, and intermediate types, respectively. In the present study, the *MyHC-IIx* expression in the LT muscle was upregulated in the CRM group, while *MyHC-IIb* expression also displayed an upregulating trend when pigs were supplemented with FCRM relative to that in the CON group. Previous studies indicated that muscle fibers are not static structures that can adapt quickly to environmental changes, such as alterations in nutritional input [49,50]. However, the upregulated glycolytic fiber types (*MyHC-IIx* in the CRM group and *MyHC-IIb* in the FCRM group) may influence the oxidative capacity and fast-to-slow fiber type transformation in the LT muscle of pigs, which needs further research to elucidate the exact mechanism.

## 5. Conclusions

According to the results gathered in the present study, corn–soybean based diets partially replaced by CRM and FCRM can improve carcass traits and enhance the serum LDL-C level without affecting the growth performance and diarrhea rate of Huanjiang mini-pigs. Additionally, dietary CRM and FCRM supplementation improved the meat quality of Huanjiang mini-pigs by enhancing meat color, intramuscular fat, and fatty acid composition, while FCRM had better effects than CRM. Thus, CRM and FCRM would be a cost-effective feeding strategy for the development of domestic pig production.

## Figures and Tables

**Table 1:** Composition and nutrient levels of the experimental diets (air-dried, %).

Ingredients	Groups		
	CON	CRM	FCRM
Corn	58.00	55.10	55.10
Soybean meal	17.65	16.77	16.77
Wheat bran	12.80	12.16	12.16
Rice bran meal	8.30	7.89	7.89
CRM	0.00	5.00	0.00
FCRM	0.00	0.00	5.00
Lysine	0.20	0.19	0.19
Threonine	0.05	0.05	0.05
Limestone powder	0.75	0.71	0.71
Dicalcium phosphate	0.95	0.90	0.90
NaCl	0.30	0.29	0.29
Premixes ^1^	1.00	0.95	0.95
Total	100.00	100.00	100.00
Nutrient level ^2^			
Digestible energy (MJ/kg)	16.99	17.13	17.07
Crude protein	16.00	15.50	15.60
Crude fiber	4.72	4.59	4.59
*L*-Lysine	0.86	0.83	0.83
SID Methionine + Cysteine	0.55	0.53	0.54
SID Threonine	0.58	0.56	0.57
SID Tryptophan	0.17	0.17	0.17
Calcium	0.60	0.60	0.60
Phosphorus	0.50	0.50	0.54

^1^ Providing the following amounts of vitamins and minerals per kilogram of a complete diet on an as-fed basis: 16,087.9 IU, vitamin A; 4949.97 IU, vitamin D_3_; 39.60 IU, vitamin E; 4.95 mg, vitamin K_3_; 4.95 mg, vitamin B_1_; 12.37 mg, vitamin B_2_; 3.96 mg, vitamin B_6_; 0.037 mg, vitamin B_12_; 39.60 mg, Cu as CuSO_4_; 49.50 mg, Fe as FeSO_4_; 29.70 mg, Zn as ZnSO_4_; 19.80 mg, Mn as MnSO_4_; 39.60 µg, I as KI; 39.60 µg, Se as Na_2_SeO_3_; 19.80 µg, Co as CoSO_4_; 0.30 mg, biotin; 24.75 mg, pantothenic acid; 2.47 mg, folic acid; 49.50 mg, niacinamide. ^2^ Data are the results of chemical analyses conducted in triplicate. CON, control; CRM, cassava residue meal; FCRM, fermented cassava residue meal.

**Table 2:** Primers used for RT-PCR in this study.

Gene Name	Primers (5′–3′)	Product Size (bp)
*β-actin*	F: CGTTGGCTGGTTGAGAATC	132
	R: CGGCAAGACAGAAATGACAA	
*MyHC-I*	F:GGCCCCTTCCAGCTTGA	63
	R: TGGCTGCGCCTTGGTTT	
*MyHC-IIa*	F: TTAAAAAGCTCCAAGAACTGTTTCA	100
	R: CCATTTCCTGGTCGGAACTC	
*MyHC-IIx*	F: AGCTTCAAGTTCTGCCCCACT	76
	R: GGCTGCGGGTTATTGATGG	
*MyHC-IIb*	F: CACTTTAAGTAGTTGTCTGCCTTGAG	80
	R: GGCAGCAGGGCACTAGATGT	

*MyHC*, myosin heavy chain.

**Table 3:** Effects of dietary CRM and FCRM supplementation on the growth performance and diarrhea rate of Huanjiang mini-pigs.

Item	CON	CRM	FCRM	SEM	*p*-Values
Initial BW (kg)	8.85	8.94	8.88	0.25	0.968
Final BW (kg)	16.70	16.24	16.50	0.57	0.867
ADG (kg)	0.28	0.27	0.28	0.01	0.884
ADFI (kg)	0.85	0.84	0.81	0.02	0.363
F/G	3.07	3.23	2.97	0.15	0.508
Diarrhea rate (%)	13.01	12.36	10.11	1.58	0.423

Data are presented as means with their SEM (*n* = 8). CON, control group; CRM, cassava residue meal group; FCRM, fermented cassava residue meal group; ADG, average daily gain; ADFI, average daily feed intake; F/G, fed-to-gain ratio.

**Table 4:** Effects of dietary CRM and FCRM supplementation on serum lipid indicators of Huanjiang mini-pigs.

Item	CON	CRM	FCRM	SEM	*p*-Values
TG (mmol/L)	1.09	1.17	1.21	0.17	0.872
TC (mmol/L)	2.12 ^b^	2.43 ^ab^	2.61 ^a^	0.10	0.023
CHE (g/L)	720.25 ^a^	775.00 ^a^	564.86 ^b^	39.95	0.009
HDL-C (mmol/L)	0.64	0.76	0.72	0.04	0.211
LDL-C (mmol/L)	1.10 ^b^	1.44 ^a^	1.60 ^a^	0.11	0.018

Data are presented as means with their SEM (*n* = 8). Mean values without a common superscript letter in the same row are significantly different (*p* < 0.05). CON, control group; CRM, cassava residue meal group; FCRM, fermented cassava residue meal group; TG, triglyceride; TC, total cholesterol; CHE, cholinesterase; HDL-C, high-density lipoprotein-cholesterol; LDL-C, low-density lipoprotein-cholesterol.

**Table 5:** Effects of dietary CRM and FCRM supplementation on carcass traits of Huanjiang mini-pigs.

Item	CON	CRM	FCRM	SEM	*p*-Values
Live body weight, kg	17.47	16.96	17.44	0.59	0.795
Hot carcass weight, kg	12.75	11.70	12.06	0.40	0.303
Carcass yield, %	74.87 ^a^	69.08 ^b^	68.97 ^b^	1.17	0.003
Lean meat percentage, %	29.61	29.94	28.73	0.45	0.204
Fat percentage, %	11.27	9.53	9.59	0.83	0.271
Bone percentage, %	15.60	15.96	16.68	0.73	0.578
Loin-eye area, cm^2^	7.69	7.62	6.71	0.43	0.228
Back fat, cm	1.98 ^a^	1.49 ^b^	1.52 ^b^	0.11	0.009

Data are presented as means with their SEM (*n* = 8). Mean values without a common superscript letter in the same row are significantly different (*p* < 0.05). CON, control group; CRM, cassava residue meal group; FCRM, fermented cassava residue meal group.

**Table 6:** Effects of dietary CRM and FCRM supplementation on meat quality of Huanjiang mini-pigs.

Item	Con	CRM	FCRM	SEM	*p*-Values
pH_45 min_	6.41	6.23	6.32	0.06	0.204
pH_24 h_	6.04	5.56	5.92	0.15	0.090
a* value	18.71 ^b^	23.23 ^a^	22.05 ^a^	0.95	0.013
b* value	6.86	6.75	7.07	0.54	0.915
L* value	54.33 ^a^	47.85 ^b^	47.99 ^b^	1.89	0.039
Cooking yield (%)	70.94	76.24	76.03	2.40	0.250
IMF	4.93 ^b^	6.33 ^a^	6.46 ^a^	0.26	0.013

Data are presented as means with their SEM (*n* = 8). Mean values without a common superscript letter in the same row are significantly different (*p* < 0.05). CON, control group; CRM, cassava residue meal group; FCRM, fermented cassava residue meal group; a*, redness; b*, yellowness; L*, lightness; IMF, intramuscular fat.

**Table 7:** Effects of dietary CRM and FCRM supplementation on fatty acid composition in the *longissimus thoracis* muscle of Huanjiang mini-pigs.

Item (%)	CON	CRM	FCRM	SEM	*p*-Values
C12:0	0.12	0.12	0.12	0.01	0.997
C14:0	1.35	1.55	1.60	0.78	0.382
C15:0	0.03	0.03	0.03	0.00	0.986
C16:0	24.81	22.26	20.59	1.29	0.175
C17:0	0.17	0.17	0.17	0.01	0.992
C18:0	13.16	11.94	12.32	0.58	0.691
C20:0	0.18 ^a^	0.14 ^b^	0.14 ^b^	0.01	0.004
C22:0	0.01	<0.01	0.01	0.00	0.222
∑SFA ^1^	39.84 ^a^	36.21 ^ab^	34.97 ^b^	0.84	0.035
C16:1	5.86	6.53	7.12	0.61	0.826
C18:1n-9	35.89	39.68	41.81	1.26	0.155
C20:1	0.58	0.51	0.65	0.04	0.348
∑MUFA ^2^	42.33	46.72	49.28	1.30	0.083
C18:2n-6	13.30	11.69	12.08	0.64	0.576
C20:2	0.26	0.23	0.26	0.01	0.508
C18:3n-3	0.02	0.02	0.02	0.00	0.355
C20:3n-6	0.44	0.38	0.40	0.02	0.429
C20:4n-6	1.59	1.58	1.98	0.11	0.295
C22:6	0.21	0.17	0.17	0.01	0.167
∑PUFA ^3^	15.56	13.84	14.65	0.73	0.637
∑SFA/∑UFA ^4^	0.69 ^a^	0.61 ^ab^	0.55 ^b^	0.02	0.043

Data are presented as means with their SEM (*n* = 8). Mean values without a common superscript letter in the same row are significantly different (*p* < 0.05). CON, control group; CRM, cassava residue meal group; FCRM, fermented cassava residue meal group. ^1^ ∑SFA = sum of saturated fatty acid. ^2^ ∑MUFA = sum of monounsaturated fatty acid. ^3^ ∑PUFA = sum of polyunsaturated fatty acid. ^4^ ∑SFA/∑UFA = sum of saturated fatty acid/sum of unsaturated fatty acid.

## Data Availability

The datasets generated and/or analyzed during the current study are available from the corresponding author upon reasonable request.
